# Climate stability is more important than water–energy variables in shaping the elevational variation in species richness

**DOI:** 10.1002/ece3.4202

**Published:** 2018-06-11

**Authors:** Jie Gao, Yanhong Liu

**Affiliations:** ^1^ Forestry College of Beijing Forestry University Beijing China

**Keywords:** boundary constraint, elevational, environmental stability, physiological tolerance, species richness, water–energy

## Abstract

Changes in climate variables have an important impact on the prediction and protection of elevational biodiversity. Gaps exist in our understanding of the elevational distribution patterns in seed plant species richness. Our study examines the importance of climate variables in shaping the elevational variation in species richness. The importance of boundary constraint was also taken into account. Model selection based on Akaike's information criterion was used to select the best explaining climate models. Variation partitioning was used to assess the independent and joint effects of water–energy, physiological tolerance, and environmental stability variables on species richness. Our results revealed that: (a) Both raw (boundary constraint unreduced) and estimated (boundary constraint reduced) species richness showed large elevational variation, with the peak species richness seen at midelevations. The environmental variables were better at explaining the distribution pattern of species richness along the elevation, when the effect of boundary constraint was reduced; (b) the physiological tolerance and environmental stability variables explained more variation in raw and estimated species richness compared with the water–energy variables. Estimated species richness was better explained (98.6%) by the environmental variables than raw species richness (94%); (c) the water‐related variables generally had the highest independent effect on raw and estimated species richness and were dominant in shaping the elevational variation in species richness. Our findings quantify the influence of boundary constraint on the distribution pattern of species along an altitudinal gradient and compare the relative contributions of environmental stability and water–energy in explaining the altitude gradient distribution pattern of plant seed species.

## 1 INTRODUCTION

One of the central issues in community ecology is understanding the underlying mechanisms shaping the spatial variation in species richness (Francis & Currie, 2003; Lomolino, Riddle, Whittaker, & Brown, 2010; Nogues‐Bravo, Araujo, Romdal, & Rahbek, 2008). Changes in species richness along elevational gradients have proven to be useful platforms to research the effects of climate change on the mechanisms of species coexistence (Grytnes, 2003; Körner, 2000, 2007). The number of hypotheses that have been proposed to explain this has increased sharply during the past half‐century (Field, Hawkins, & Cornell, 2009; Kessler, Kluge, Hemp, & Ohlemüller, 2011; Willig, Kaufman, & Stevens, 2003). No single hypothesis can fully explain the true formation and maintenance of diversity gradients, and few empirical studies have examined these hypotheses together to explain this phenomenon (Grytnes & Beaman, 2006).

One of the main hypotheses used to explain species richness gradients is the water–energy hypothesis (Francis & Currie, 2003; Kaspari, O'Donnell, & Kercher, 2000; Wang & Fang, 2012). It predicts that regions with higher water and energy availability can hold more species, although the strength of this relationship is dependent on the spatial scale (Evans, Newson, Storch, Greenwood, & Gaston, 2008; Gaston, 2000; O'Brien, 1993). The physiological tolerance hypothesis (Connell & Orias, 1964) proposes that environments with suitable temperature conditions and water availability have a greater species richness, because more species can tolerate these conditions, whereas only a small number of species can endure more demanding environmental conditions. Many species cannot survive in extremely cold or hot environments (Jing, Ma, & Anand, 2016; Roy, Stanton, & Eppley, 1999). In addition to the above two hypotheses, Connell and Orias (1964) proposed that, with greater environmental stability, less energy is required for regulatory activities. That is, for those species able to endure the environmental challenges, more energy is allocated to net productivity, growth, and reproduction.

While climate factors can shape the elevational variation in species richness, both boundary constraint (i.e., geographical boundaries affecting the distribution of species) and distribution area can also have a significant influence. The mid‐domain effect states that if the species ranges are randomly distributed within a bounded domain, then more species ranges will overlap near the middle of the domain than at the edges. Thus, species richness will decrease from the mid‐domain to the edges. However, the relative role of boundary constraint in such patterns differs markedly depending on many factors, including water and temperature (Wang & Fang, 2012). The species–area relationship predicts that, in a relatively homogeneous area, more individuals will occur with increases in the sampling area, revealing complex ecological processes that may increase the species richness (Losos & Schluter, 2000).

In our study, we suggest that three hypotheses (i.e., water–energy, physiological tolerance, and environmental stability) can explain elevational patterns in species richness. However, each hypothesis can be interpreted to a differing degree. Our goal was to answer the following questions: (a) Do the plant species show an obvious elevational distribution pattern and do boundary constraint shape the elevational variation in species richness?; (b) can species richness patterns be explained by the three hypotheses and which is better?; and (c) which environmental variables are the most important in determining elevational patterns of species richness?

## 2 METHODS

### 2.1 Study area

The Tongbiguan Nature Reserve in Yunnan Province, China covers a total area of 73,216 ha. The average annual temperature in the region is 19.47°C, the average hours of annual sunshine are 2,357.8 h, and the average cumulative rainfall is 1,660 mm. The topography of the area is complex, with the highest elevation being 2,595 m and the lowest 210 m. The vast majority of mountains are below 2,000 m above sea level. The Reserve contains 3,767 species of wild tracheophyte belonging to 1,316 genera in 248 families.

### 2.2 Species data

Species distribution data along the elevational gradient within the reserve were compiled from the book “Scientific investigation and Research on the nature reserve of Tongbiguan in Yunnan, China” (Yang & Du, 2006). This book is the most reliable to date for cataloguing the plant species in the Reserve. The plant species diversity was systematically recorded in this book. Most species here are distributed between 200 and 2,000 m above sea level. Therefore, we divided the study area into 18 sections along this elevation gradient, with each section covering a 100‐m elevation interval, following the method used in studies on tropical communities (Sanders, 2002). We calculated empirical species richness as interpolated species richness, based on the assumption that each plant species inhabited all sampling sites between its lowest and highest recorded occurrences, regardless of whether or not it was recorded at all intermediate sites (Bhattarai, Vetaas, & Grytnes, 2004; Geml, Morgado, Semenovanelsen, & Schilthuizen, 2017; Geml et al., 2014). In this respect, we summarize the altitude distribution information for each plant and counted the number of species in each elevation section. For a very few species, the elevational distribution of the species was not clear, so we referred to the specimen collections in herbarium to determine the altitude distribution of the species.

### 2.3 Environmental data

For each 100‐m elevation interval, we used nine environmental variables to compare the relative role of water–energy, physiological tolerance, and environmental stability in explaining the spatial variation of species richness at the elevational scale and environmental correlates of seed plants (Table 1). The environmental variables were downloaded from the Worldclim‐Global Climate data base (http://www.worldclim.org/current). WorldClim is a set of global climate layers (climate grids) with a spatial resolution of 1 km^2^. The data can be used for mapping and spatial modeling in GIS or with other computer software. The water–energy variables included: annual mean temperature (TEM, K) and annual precipitation (AP, mm) (Chen et al., 2015). The physiological tolerance variables included: maximum temperature during the warmest month (MATW, K), minimum temperature during the coldest month (MITW), precipitation during the wettest month (WP, mm), and precipitation during the driest month (DP, mm) (Jing et al., 2016). The environmental stability variables included: precipitation seasonality (coefficient of variation) (SP), mean diurnal range (MDR), and temperature annual range (ART, K) (Chen et al., 2015). The surface area of each 100‐m elevation interval was obtained using interpolation tools in ArcGIS (Gao, Zhang, Luo, Lan, & Liu, 2017). The boundary constraint effect (the estimated species numbers) was calculated using the Range‐Model 5 (http://viceroy.eeb.uconn.edu/RangeModel/) (Colwell, 2008). In this model, the species richness of each 100‐m elevation can be calculated with or without the boundary effect.

**Table 1:** Summary of the separate multiple regression models for the water–energy, physiological tolerance, and environmental stability hypotheses for raw species richness and estimated species richness. The model with the lowest AICc (Akaike information criterion) was selected as the best one

Hypotheses	Predictors included in the best model (standardized coefficient)	Adjusted *R* ^2^	*p*‐Value	AICc
Raw species richness				
Water–Energy	AMT(+3.17)	0.594	<0.001	19.1
Physiological tolerance	MATW (+2.13), WP (+23.20), DP(+0.13)	0.883	<0.001	5.6
Environmental stability	SP (+0.27), MDR (−2.75)	0.863	<0.001	4.1
Estimated species richness				
Water–Energy	AMT(+4.94)	0.664	<0.001	29.2
Physiological tolerance	WP (+20.46)	0.907	<0.001	7.2
Environmental stability	SP (1.47), MDR (−3.12)	0.973	<0.001	−10.2

The variable selection was performed within each group separately.

AMT: annual mean temperature; DP: precipitation of driest month; MATW: max temperature of the warmest month; MDR: mean diurnal range; SP: precipitation seasonality (coefficient of variation); WP: precipitation of wettest month.

### 2.4 Statistical analyses

The species richness data were assumed to fit a Poisson distribution and were connected with the environmental factors by a logarithmic function (Martínez, Flores, Aragón, Otálora, & Rubio‐Salcedo, 2014; McCullagh & Nelder, 1989). In our paper, we used species density (species richness/area) to represent species richness to reduce the effect of area in each elevation section (Chen et al., 2015).We used GLM to examine the relationships between response variables and potential predictors. Adjusted *R* ^2^ was used to estimate the explanatory power of the regression models. The statistical significance of all the correlations and regressions was based on corrected degrees, calculated using a *t*‐test.

The relationships between the nine environmental variables were examined by Pearson's correlation analyses and showed a strong multicollinearity (Supporting information Figure S2). Multicollinearity is a phenomenon in which one predictor variable in a multiple regression model can be linearly predicted from the others with a substantial degree of accuracy (Heikkinen, Luoto, Kuussaari, & Poyry, 2005). We used variation and hierarchical partitioning to deal with this problem. Variation partitioning can be used to determine the relative explanatory power of the three hypotheses (water–energy, physiological tolerance, and environmental stability) for each response variable (Heikkinen et al., 2005). We used AICc (corrected Akaike information criterion; Montoya, Rodriguez, Zavala, & Hawkins, 2007) to select the best models for raw and estimated species richness, and the selected environmental variables in subsequent analyses. Variance partitioning analysis led to the following pure fractions: (a) effect of water–energy; (b) effect of physiological tolerance; and (c) effect of environmental stability; as well as the following fractions with combined variation due to the joint effects: (a) water–energy and physiological tolerance; (b) water–energy and environmental stability; (c) physiological tolerance and environmental stability; (d) three groups of explanatory variables; and (e) unexplained variation.

We used hierarchical partitioning in the R 4.0 statistical package (hier.part) (Mac‐Nally & Walsh, 2004) to identify the contribution of selected variables on the raw and estimated species richness. The partitioning used linear regression and *R* ^2^ as the goodness‐of‐fit measure. The significance of the independent effect of variables was tested by a randomization routine, which yielded *Z*‐scores for the generated distribution of randomized independent contributions, with the significance based on an upper 0.95 confidence limit. As a result, hierarchical partitioning provides, for each explanatory variable separately, an estimate of the independent and joint contribution with all other variables (Chen et al., 2015). Most statistical analyses were carried out using SAM (Spatial Analysis in Macroecology) and the R 4.0 statistical package.

## 3 RESULTS

**Figure 1:**
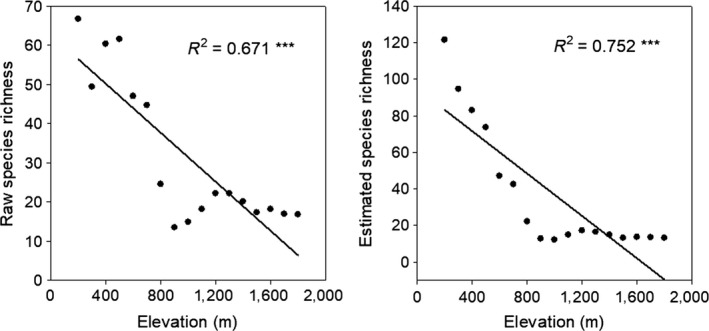
The elevational variation in raw and estimated species richness. *R* ^2^ was used to estimate the explanatory power of the regression models. * represents the significant level. *p < 0.05, **p < 0.01, ***p < 0.001

**Figure 2:**
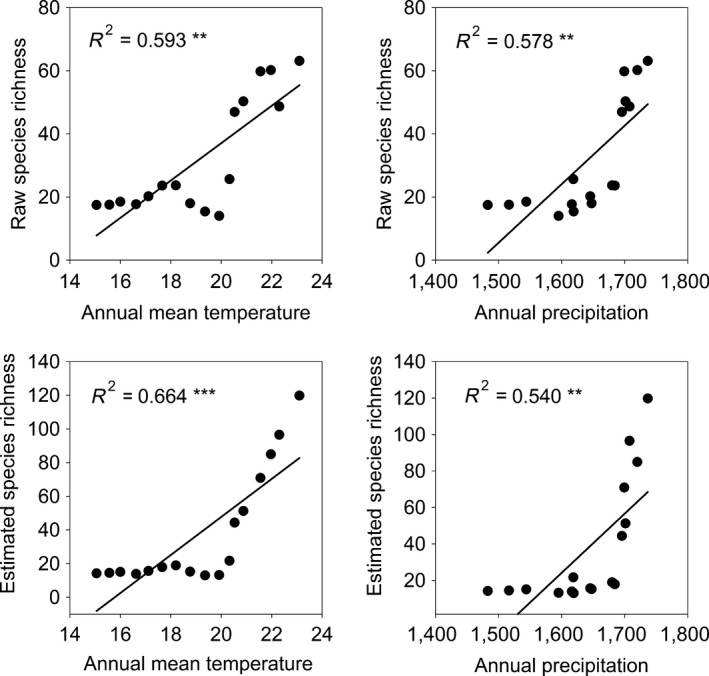
Relationships between the raw and estimated species richness with the annual mean temperature and annual precipitation. *R* ^2^ was used to estimate the explanatory power of the regression models. * represents the significant level. *p < 0.05, **p < 0.01, ***p < 0.001

The boundary constraint had a significant effect on elevational variation in species richness. Both raw and estimated species richness showed large elevational variation, with the peak richness seen at midelevations, peak species density seen at low elevations (Supporting information Figure S1; Figure 1). Generally, raw and estimated species richness showed more significant correlations with most climatic variables than MDR and ART according to the *t*‐test (Supporting information Table S1; Figure 2).

Table 1 shows the selected predictors for raw and estimated species richness of seed plants from the three variable groups. The physiological tolerance and environmental stability variables explained more variation in raw and estimated species richness as compared with the water–energy variables. For raw species richness, the explanatory power of the selected physiological tolerance variables (MATW, WP, and DP, 88.3%) was very similar to that of environmental stability variables (SP and MDR, 86.3%), but much higher than that of the selected water–energy variable (AMT, 59.4%). For estimated species richness, the explanatory power of the selected physiological tolerance variables (WP, 90.7%) was very similar to that of environmental stability variables (SP and MDR, 97.3%) and much higher than that of the selected water–energy variable (AMT, 66.4%). Compared to raw species richness, estimated species richness was better explained by the environmental variables.

**Figure 3:**
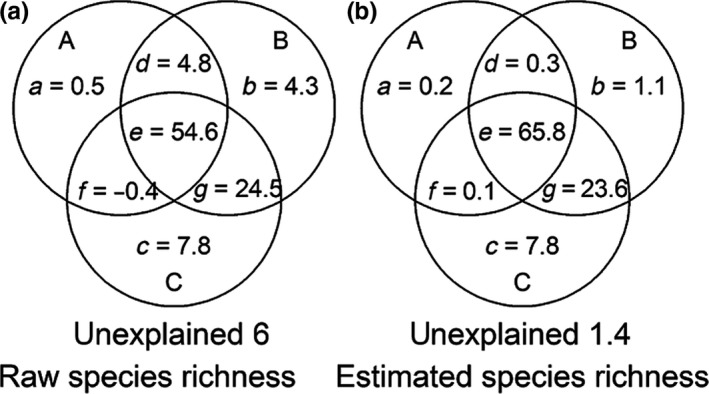
Results of variation partitioning for raw and estimated species richness in terms of the fractions of variation explained. A: water–energy B: physiological tolerance C: environmental stability variables. The variation in species richness is explained by the three groups of explanatory variables (A, B, C), and unexplained is the undetermined variation. a, b, and c are the unique effects of water–energy, physiological tolerance, and environmental stability. d, e, f, and g are fractions indicating their joint effects

Results of the variation partitioning showed that physiological tolerance and environmental stability variables had the highest explanatory power for raw species richness (24.5%) and estimated species richness (23.6%) (Figure 3). For raw and estimated species richness, the independent effects of environmental stability were larger (7.6%, 7.8%) than for water–energy (0.5%, 0.2%) and physiological tolerance (4.3%, 1.1%). Estimated species richness could be well‐explained by the environmental variables (98.6%). The environmental variables were better able to explain the distribution pattern of species richness along the elevation gradient when the effect of boundary constraint was considered.

**Figure 4:**
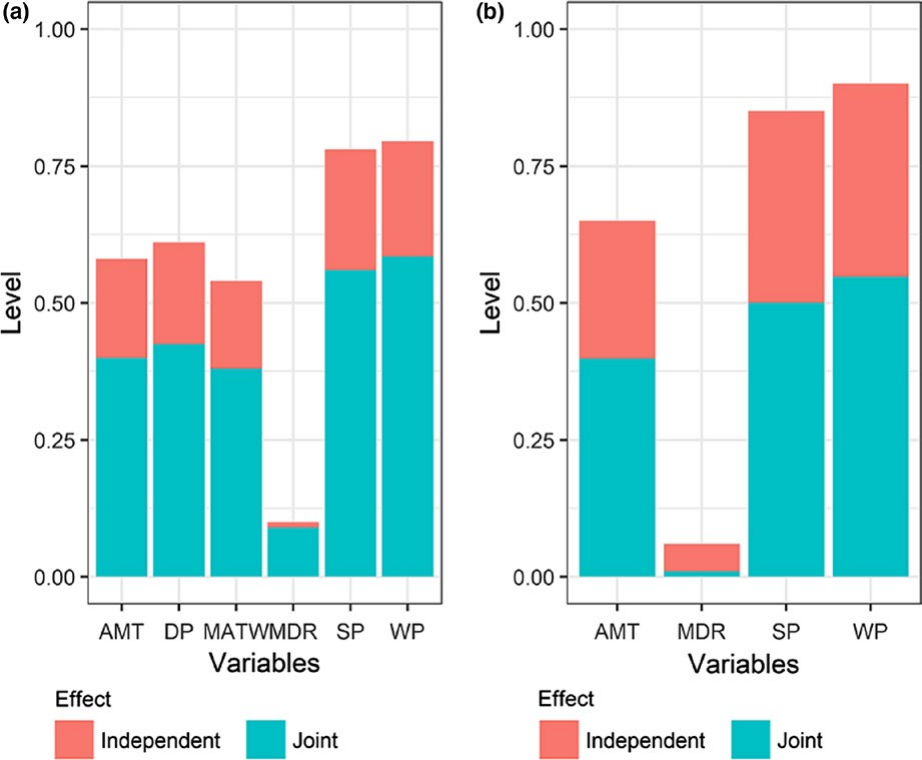
Results of the hierarchical partitioning for raw and estimated species richness in terms of the independent and joint effects of the predictors. Level: the degree of interpretation of species diversity by each variable; AMT: annual mean temperature; MATW: max temperature during the warmest month. WP: precipitation during the wettest month. DP: precipitation during the driest month. SP: precipitation seasonality (coefficient of variation). MDR: mean diurnal range

In the hierarchical partitioning, all the temperature‐related variables generally had the lowest independent effect on raw and estimated species richness (Figure 4). For raw and estimated species richness, SP and WP had the highest independent effect, while MDR had the lowest dependent contribution to the variation.

## 4 DISCUSSION

### 4.1 Elevational patterns: role of boundary constraint

Many studies have found that a number of species show different distribution patterns along elevational gradients (Gao et al., 2017; Sanders, 2002; Wang, Tang, & Fang, 2003). In our study, seed plant species richness peaked at midelevations (Supporting information Figure S1).This species richness pattern was similar to that observed in other places (Geml et al., 2017; Sanders, 2002) but differs from the pattern of monotonal decrease with increasing elevation in temperate mountains (Bahram, Polme, Koljalg, Zarre, & Tedersoo, 2012; Nouhra, Urcelay, Longo, & Fontenla, 2012). Midelevations offered a wider variety of ecological strategies, which ensured lots of plant species can pass the extreme weather more easily (Jing et al., 2016). It is widely accepted that: with increasing area, habitats become more complex and can provide a wider variety of ecological niches and environmental resources, thereby potentially increasing species diversity (Rahbek, 2005). In our results, when we used the species density to represent species richness, plant species diversity decreased significantly with elevation gradient (Figure 1). The area contains many complex ecological processes which decrease rapidly with increasing altitude, and results in fewer plant species (MacArthur, 1972; Rahbek, 2005).

Our results supported the finding that boundary constraint has a relatively significant effect on elevational variation in species richness, which was similar to that found by Colwell (2008) and Wang and Fang (2012). When we reduced the effect of boundary constraint, plants showed a more significant gradient along the elevation (Figures 1 and 3). The limits that boundary constraint places on species distribution can make the distribution range of different species in the border area smaller. However, the degree of overlap is larger in the central area, so that the species diversity in the central area is relatively high (Colwell, 2008). Species richness patterns in various vascular plant groups have been shown to exhibit varying degrees of boundary constraint effect on Mt. Kinabalu and Neotropical mountains (Cardelus, Colwell, & Watkins, 2006), although boundary constraint alone cannot fully explain the observed patterns (Cardelus et al., 2006; Colwell, 2008). This finding is markedly different from patterns found in Japan, where boundary constraint was found to explain most of the richness patterns (Miyamoto, Nakano, Hattori, & Nara, 2014).

### 4.2 Effects of water–energy, physiological tolerance and environmental stability

Environmental factors are known to change along elevation gradients and plant species found at different elevations are differentially adapted to these varying conditions (Wang & Fang, 2012). However, the present species distribution patterns could be better understood by the combination of environmental factors and geometric constraints. In most organisms, the environmental variables most commonly related to species richness are generally measures of water and energy availability (O'Brien, 1993, 1998; Rahbek, 2005). Some studies have addressed the physiological tolerance (Jing et al., 2016; Spasojevic, Grace, Harrison, & Damschen, 2014) and environmental stability (Connell & Orias, 1964) hypotheses as explanations for the elevational patterns and spatial variation in species richness.

Our results showed that environmental variables related to water–energy, physiological tolerance and environmental stability are important factors for explaining the elevational pattern of raw and estimated species richness. Physiological tolerance and environmental stability were most closely related to the elevational pattern of raw and estimated species richness, with water–energy accounting for smaller but considerable contribution (Figure 3). Roy et al. (1999) and Kluge, Kessler, and Dunn (2006) suggested that extreme temperatures limit species richness at lower and higher elevations. Plants communities at mid‐elevation should contain more species, because more species can tolerate benign conditions. In contrast, a smaller number of species can withstand the challenges of more demanding environmental conditions. However, they provided no direct evidence for that hypothesis. Until now, few empirical studies have demonstrated a link between extreme temperatures and plant richness along an elevational gradient (Jing et al., 2016). Connell and Orias (1964) found that environmental stability best explained the level of ecological community diversity and found that, with greater environmental stability, less energy is required for regulatory activities (i.e., more energy is allocated for net productivity, growth, and reproduction). To our knowledge, our results provide the first empirical evidence of a link between environmental stability and plant richness along an elevational gradient. There is still, however, 1.4% (Figure 3b) of the variation that cannot be explained by above three hypotheses. We acknowledged that geographic speciation events and spatially constrained evolutionary histories could be responsible for the remaining unexplained spatial variation (Chen et al., 2015).

The interaction between water and energy is crucial for species richness (O'Brien, 1993). Our results showed that water‐related variables were dominant in shaping the elevational variation in species richness (Figure 4). This may be because high temperatures can lead to high evapotranspiration and increased water stress. These environmental filters can exclude drought intolerant species (Bhattarai et al., 2004).

This is the first empirical study investigating the explanation of water–energy, physiological tolerance, and environmental stability on plant species richness along elevation gradients. Climate and geographical factors appear to be important in shaping the distribution of plant species. It would be interesting to further explore the relationship between elevation and the distribution of other species (e.g., animals and insects), while considering the influence of climate stability and the effects of geometric constraints. We expect that climate stability and geometric constraints will be among the important influencers of species richness along elevational gradients.

## CONFLICT OF INTEREST

None declared.

## AUTHOR CONTRIBUTIONS

J Gao and YH Liu designed the study and collected the data. J Gao performed analyses and led the writing. J Gao and YH Liu contributed substantially to revisions.
