# Supplementary material for: Climate stability is more important than water–energy variables in shaping the elevational variation in species richness
Source: Ecol Evol. 2018 Jun 11;8(14):6872–9. doi: 10.1002/ece3.4202 (PMC6065338; doi:10.1002/ece3.4202)
Supplement: Supplementary file 1 [file ECE3-8-6872-s001.docx]

**Appendix**

Table S1 Statistics of univariate regressions on raw and estimated species richness against the environmental variables.

| Variables | Coefficient ± SD | |  | *t* | |  | *R*^2^ | |
| --- | --- | --- | --- | --- | --- | --- | --- | --- |
|  | Raw  richness | Estimated richness |  | Raw  richness | Estimated richness |  | Raw  richness | Estimated richness |
|  |  |  |  |  |  |  |  |  |
| AMT | 3.166±0.68 | 4.936±0.91 |  | 4.686** | 5.439*** |  | 0.567 | 0.641 |
| AP | 9.066±2.00 | 12.939±3.08 |  | 4.525** | 4.198** |  | 0.549 | 0.510 |
| MATW | 3.821±0.89 | 5.931±1.22 |  | 4.308*** | 4.871*** |  | 0.523 | 0.587 |
| MITC | 0.413±0.12 | 0.628±0.18 |  | 3.378** | 3.563** |  | 0.394 | 0.423 |
| WP | 12.814±1.79 | 20.462±1.69 |  | 7.174*** | 12.120*** |  | 0.759 | 0.901 |
| DP | 13.312±2.79 | 17.950±4.60 |  | 4.77*** | 3.902** |  | 0.576 | 0.471 |
| SP | 11.112±1.61 | 17.506±1.77 |  | 6.901*** | 9.903*** |  | 0.744 | 0.859 |
| MDR | -7.801±7.86 | -11.491±11.77 |  | -0.907 | -0.976 |  | 0.032 | 0.012 |
| ART | 11.281±5.82 | 22.960±8.25 |  | 2.455* | 2.783* |  | 0.239 | 0.297 |

Fig.S1 The elevational variation in raw species richness.

Fig. S2 Pearson's correlation coefficients between environmental variables using R package. The amount of blue or red in the circles indicates the magnitude of the correlation of the factors. Blue signifies a positive correlation, red signifies a negative correlation.
